# A comparison of methods for monitoring photon beam energy constancy

**DOI:** 10.1120/jacmp.v17i6.6454

**Published:** 2016-11-08

**Authors:** Song Gao, Peter A. Balter, Mark Rose, William E. Simon

**Affiliations:** ^1^ Department of Radiation Physics The University of Texas MD Anderson Cancer Center Houston TX USA; ^2^ Sun Nuclear Corporation Melbourne FL USA

**Keywords:** flattening filter‐free beam, flatness, ionization chamber array, energy change, quality assurance

## Abstract

In extension of a previous study, we compared several photon beam energy metrics to determine which was the most sensitive to energy change; in addition to those, we accounted for both the sensitivity of each metric and the uncertainty in determining that metric for both traditional flattening filter (FF) beams (4, 6, 8, and 10 MV) and for flattening filter‐free (FFF) beams (6 and 10 MV) on a Varian TrueBeam. We examined changes in these energy metrics when photon energies were changed to ±5% and ±10% from their nominal energies: 1) an attenuation‐based metric (the percent depth dose at 10 cm depth, PDD(10)) and, 2) profile‐based metrics, including flatness (Flat) and off‐axis ratios (OARs) measured on the orthogonal axes or on the diagonals (diagonal normalized flatness, $F_{\text{DN}}$). Profile‐based metrics were measured near $d_{\text{max}}$ and also near 10 cm depth in water (using a 3D scanner) and with ionization chamber array (ICA). PDD(10) was measured only in water. Changes in PDD, OAR, and $F_{\text{DN}}$ were nearly linear to the changes in the bend magnet current (BMI) over the range from −10% to +10% for both FF and FFF beams: a ±10% change in energy resulted in a ±1.5% change in PDD(10) for both FF and FFF beams, and changes in OAR and $F_{\text{DN}}$ were >3.0% for FF beams and >2.2% for FFF beams. The uncertainty in determining PDD(10) was estimated to be 0.15% and that for OAR and $F_{\text{DN}}$ about 0.07%. This resulted in minimally detectable changes in energy of 2.5% for PDD(10) and 0.5% for OAR and $F_{\text{DN}}$. We found that the OAR‐ or FDN‐ based metrics were the best for detecting energy changes for both FF and FFF beams. The ability of the OAR‐based metrics determined with a water scanner to detect energy changes was equivalent to that using an ionization chamber array. We recommend that OAR be measured either on the orthogonal axes or the diagonals, using an ionization chamber array near the depth of maximum dose, as a sensitive and efficient way to confirm stability of photon beam energy.

PACS number(s): 87.55.Qr, 87.56.Fc

## I. INTRODUCTION

Quality assurance (QA) for medical linear accelerators (linacs) is done to ensure that the machine characteristics have not changed from their baselines acquired at the time of commissioning or from the model in the treatment planning system. For photon beams, this includes verifying the consistency of the beam profile (flatness and symmetry) as a verification of beam steering and checking percent depth dose (PDD) or tissue maximum ratio (TMR)^(1)^ as a verification of the photon beam energy.

A recent study^(2)^ indicated that energy change (generated by the change the bending magnet current [BMI]) is better observed as changes in beam profile than as changes in PDD in Varian C‐series linacs. That study showed that the diagonal normalized flatness, $F_{\text{ND}}$ was the best metric for monitoring changes in photon beam energy. That finding was confirmed by a very recent study by Goodall et al.^(3)^ of Elekta linacs.

This work is an extension of an earlier study^(2)^ that was based on data collected from a decommissioned analog clinical linac which had only flattening filter (FF) beams. The design of the linac combined with time constraints due to the linac's removal limited the amount and quality of the data that could be acquired. We have extended the previous study in three ways: We have 1) studied more beam energies including FFF beams; 2) acquired more and higher quality data enabling better analysis; 3) estimated the sensitivity of each metric based on its relative changes compared to its measurement uncertainty. We measured the changes in beam profiles and PDDs as a function of photon energy for both FF and flattening filter‐free (FFF) photon beams on a digital linac (TrueBeam, Varian Medical Systems, Palo Alto, CA). The digital architecture of the TrueBeam allowed the beam parameters associated with each beam energy to be saved and reloaded as needed, enabling us to sample a large number of energies with different measurement setups without interfering with the clinically commissioned beams. The concept that photon beam energy variations can be monitored by beam profiles comes from the observation that the angular distribution of intensity and energy spectrum of a photon beam created via Bremsstrahlung interactions are a function of the incident electron energy.^4^, ^5^ The absolute energy spectrum is not needed for routine QA, and it is sufficient to monitor changes in the beam profile to monitor changes in the beam energy. Energy stability has traditionally been monitored by using attenuation‐based metrics such as the PDD or tissue maximum ratio, but these metrics are less sensitive to energy change than are changes in beam profile.^2^, ^3^

The goal of this study was to determine which energy metric and equipment combination was most likely to find a real change in beam energy during routine QA, based on both the change in that metric with energy and the measurement uncertainty of the metric.

## II. MATERIALS AND METHODS

### A. Comparison of various energy metrics

Percent depth dose is an attenuation‐based metric traditionally used for monitoring beam energy.^1^, ^6^ For simplicity, the PDD is tracked at a fixed combination of depth, field size, and source‐to‐surface distance (SSD); a 10 cm depth in a $10\times 10\;{\text{cm}}^{2}$ field at an SSD of 100 cm is the most common reference condition. The PDD(10) is defined as the ratio of the dose at a depth of 10 cm to the dose at the depth of maximum dose $\left(d_{\text{max}}\right)$ for a 100 cm SSD at a specified field size.

In this study, we measured PDD and beam profiles as a function of change in beam energy for several FF and FFF photon beams where the beam's energy was adjusted by changing the BMI over a range of ±10% from its nominal values. For FF and FFF beams, we compared the changes in OAR and $F_{\text{DN}}$ to the changes in PDD as a function of change in photon beam energy. For FF beams, we also examined flatness (Flat) (Eq. 1). Profiles were measured both in water with a 3D scanning (3DS) system and with an ionization‐chamber array (ICA) at depths near $d_{\text{max}}$ and near 10 cm. PDD was measured in water. We used repeated measurements with similar equipment to estimate the uncertainty in these determinations.

We compared PDD(10) at different BMI settings to PPD(10) at the nominal BMI setting for each beam to determine its sensitivity to energy changes. For profile‐based energy metrics, we calculated and compared flatness (Flat) as defined in the report of AAPM Task Group 45^(7)^ (Eq. 1) and off‐axis ratio as defined below (Eq. 2). Flat is defined as the maximum variation over the central 80% of the full width at half maximum (FWHM) of the profile in a plane transverse to the beam axis:

(1) $$Flat=\frac{R_{max}-R_{min}}{R_{max}+R_{min}}\times 100\%$$

where $R_{max}$ and $R_{min}$ are the maximum and minimum dose values measured along the cardinal orthogonal axes of the beam. Flat is directly reported by software package included with the IC Profiler.^(2)^ For this study we have defined the off‐axis ratio (OAR) to be the ratio of the average measurements at a fixed distance from the beam central axis (CAX) to the measurement at the CAX:

(2) $$OAR=\frac{(\Sigma_{i=1}^{4}R_{d}^{i})/4}{R_{CAX}}\times 100,$$

where $R_{CAX}$ is the measured value at the CAX and $R_{d}^{i}$ are the measured values at a fixed position (distance approximately 80% of the field size from CAX) on the orthogonal axes. The effects of variations in beam steering are minimized by averaging the values sampled along each axis. The definition of OAR is similar to the diagonal normalized flatness $\left(F_{\text{DN}}\right)$ defined in previous studies,^2^, ^3^ except that the $F_{\text{DN}}$ metric was along the diagonal axes.

## B. Equipment

### B.1 Water scanning system

PDD and beam profile metrics were measured in water with a small‐volume ionization chamber (CC04, IBA Dosimetry GmbH, Schwarzenbruck, Germany) and a commercial 3D water scanner (3DS) (Sun Nuclear Corp., Melbourne, FL). All measurements were done with −300 V on the central electrode, and no corrections were made for polarity or collection efficiency. All depths are to the central axes of the ion chamber.

### B.2 Ionization chamber array

Beam profiles were also measured with a commercially available ionization chamber array (ICA) (PROFILER, Sun Nuclear Corp.). The ICA has 251 ionization chambers (volume of 0.05 cm^3^) located on the x‐, y‐, negative diagonal, and positive diagonal axes. The chamber spacing is 0.5 cm on the x‐ and y‐axes and 0.71 cm on the diagonal axes, giving a measurement length of 32 cm on the orthogonal and 45 cm on the diagonal axes. The effective measurement depth is equivalent to 0.9 cm of water. A wide field calibration technique^8^, ^9^ was used to normalize the ICA before the beam profile measurements. This correction is slightly energy‐dependent and was done for the nominal energy of all FF beams; for FFF beams, we used the array normalization from the flattened beam with the same nominal energy.

### B.3 Linear accelerator

**Figure 1:**
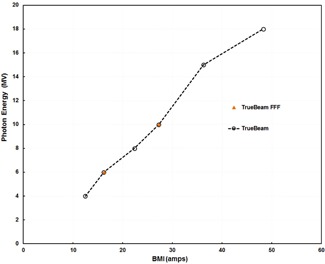
Change in photon beam energy with change in bending magnet current (BMI) for Varian TrueBeam linear accelerators. (Presented with permission from Varian Medical Systems.)

Measurements were done on a Varian TrueBeam linac (Varian Medical Systems) with 4, 6, 8, and 10 MV FF beams as well as 6 and 10 MV FFF beams. On this platform, changes in photon energy are nearly linearly proportional to changes in BMI from 4 to 15 MV (D. Pawlak, Varian Medical Systems, personal communication, December 27, 2013) (Fig. 1). Each beam tune the BMI was set to achieve the desired change in energy and the other beam parameters were tuned to achieve symmetric beams at the nominal dose rate as was done in the previous study.^(2)^ We were able to obtain stable dose rates with the BMIs adjusted up to ±10% from their nominal values. By taking advantage of the digital architecture of the TrueBeam linac, we were able to create beam tunes for each BMI setting and save the beam parameters in a file, which enabled us to load each beam tune for various measurement setups.

### C. Measurement of PDD and profile

Percent depth doses and profiles (PDDs) were scanned by using a 3DS with an SSD of 100 cm, and PDD data were acquired for three field sizes: $5\times 5\;{\text{cm}}^{2},10\times 10\;{\text{cm}}^{2}$, and $30\times 30\;{\text{cm}}^{2}$. Beam profiles in the cross‐plane (x‐) and in‐plane (y‐) were scanned for a $30\times 30\;{\text{cm}}^{2}$ field at a 10 cm depth for all beams; beam profiles were also scanned at $d_{\text{max}}$ for FFs. Manufacturer‐supplied software (SNC Dosimetry version 3.2.1) was used to calculate PDD and Flat. We manually calculated OARs from field size of $30\times 30\;{\text{cm}}^{2}$ profiles at distances of 80% of the field width at the plane of measurement.

Beam profiles were also measured by using an ICA for a $30\times 30\;{\text{cm}}^{2}$ field. For all measurements, the top of the device was placed at a 100 cm SSD. Profiles were measured with no additional buildup material on top of the device for FF and FFF beams. FF profiles were also acquired with an additional 9 cm of solid water on top of the device to give an equivalent depth of 9.9 cm. Manufacturer software (Profiler version 3.0) was used to calculate Flat for the FF. For all beams, OARs were calculated manually at distances of 80% of the field size, giving a measurement geometry equivalent to the water measurements. Diagonal normalized flatness, $F_{\text{DN}}$, was also calculated manually in this study, but it has been added as a reported metric in the manufacturer's newer version software.

### D. Minimal detectable changes in energy

We determined that the minimal detectable energy change for each metric would be when that metric changed by at least twice the uncertainty in that metric (2σ). This gives us a 95% confidence level in the detection of the energy change. We determined that change in metric for each energy metric (PDD, Flat, OAR, and $F_{\text{DN}}$) by calculating the difference between each metric at the nominal energy to that at the modified energies. We estimated the uncertainties of the measurement data acquired with the 3DS and the ICA by performing repeated measurements at the nominal energies and determining the standard deviation (σ) of that metric. We performed PDD and profile measurements five times for 6 MV FF, 10 MV FF, 6 MV FFF, and 10 MV FFF beams with the 3DS and, for the profiles only, with the ICA. For each of these five determinations, the equipment setup was repeated to ensure that equipment setup uncertainty was included. The standard deviation (σ) was calculated for each energy metric.

## III. RESULTS

### A. Changes in PDD and measurement uncertainty

**Figure 2:**
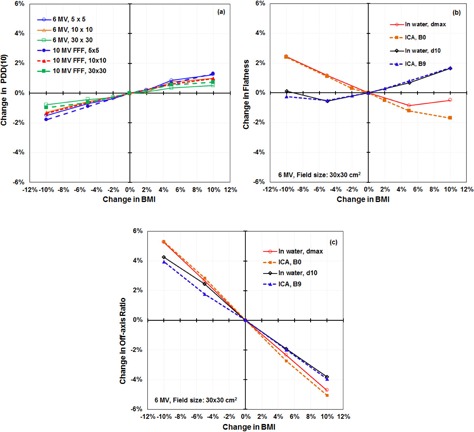
Change in energy metrics as a function of bending magnet current (BMI) for 6 MV flattened photon beams: (a) percent depth dose at 10 cm depth [PDD(10)] for different field sizes; (b) changes in flatness (Flat) in water at $d_{\text{max}}$ and depth of 10 cm $\left(d_{10}\right)$ and with an ionization chamber array (ICA) with no additional buildup $\left(B_{0}\right)$ or 9 cm solid water buildup $\left(B_{9}\right)$; (c) the diagonal normalized flatness $\left(F_{\text{DN}}\right)$ evaluated in water and with an ICA with the same setup as in (b). FFF=flattening filter−free.

We measured PDD(10) as a function of field size and BMI for FF and FFF beams. From these data, we calculated the changes in PDD(10) as a function of BMI and found them to be linear (Fig. 2(a)). As expected, changes were greatest for the smallest field size but were within 0.23%±0.12% of changes with the 10×10 field size. Changes in the PDDs as a function of BMI at other depths (e.g., 20 cm) were similar (0.11%±0.11%) to changes in PDD(10); thus we reported and analyzed only data at the 10 cm depth. Because a $10\times 10\;{\text{cm}}^{2}$ field is the standard size for monthly energy checks, we present only comparisons for the $10\times 10\;{\text{cm}}^{2}$ field (Table 1). We estimated the uncertainty in PDD measured in water with the CC04 chamber and the 3DS tank by measuring it five times for each beam but only at its nominal energy. For each set of beams we independently setup the 3DS to include setup uncertainties. For each energy we calculated the standard deviation of the PDD(10) (Table 2). We noted that this value is beam‐independent at 0.15% of the PDD(10) for that beam.

**Table 1:** Change in percent depth dose at depth of 10 cm in water (PDD(10)) as a function of bending magnet current (BMI) for $10\times 10\;{\text{cm}}^{2}$ fields for each beam at nominal energy

*Change in BMI*	*4 MV Δ% (PDD)*	*6 MV Δ% (PDD)*	*8 MV Δ% (PDD)*	*10 MV Δ% (PDD)*	*6 MV FFF Δ% (PDD)*	*10 MV FFF Δ% (PDD)*
10%	1.1 (63.5)	1.0 (67.2)	0.9 (71.4)	0.9 (74.4)	1.3 (64.3)	1.0 (71.7)
5%	0.6 (63.0)	0.8 (67.0)	0.6 (71.1)	0.6 (74.1)	0.7 (63.7)	0.6 (71.3)
0%	0.0 (62.4)	0.0 (66.2)	0.0 (70.5)	0.0 (73.5)	0.0 (63.0)	0.0 (70.7)
−5%	−0.6(61.8)	−0.5(65.7)	−0.5(70.0)	−0.6(72.9)	−0.5(62.5)	−0.6(70.1)
−10%	−1.1(61.3)	−1.3(64.9)	−1.2(69.3)	−1.2(72.3)	−1.3(61.7)	−1.4(69.3)

Numbers in parentheses are the reference PDD(10) values.

FFF=flattening filter−free.

**Table 2:** Standard deviation (σ) of percent depth dose at depth of 10 cm in water (PDD(10)) for a $10\times 10\;{\text{cm}}^{2}$ field for each beam at nominal energy

*Energy*	*4 MV*	*6 MV*	*8 MV*	*10 MV*	*6 MV FFF*	*10 MV FFF*
σ	0.14%	0.14%	0.15%	0.15%	0.15%	0.14%

FFF=flattening filter−free.

### B. Changes in profile‐based metrics and measurement uncertainties

#### B.1 Flatting filter beams

**Figure 3:**
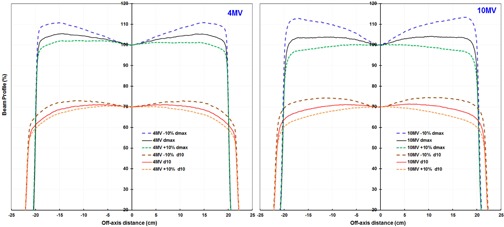
Changes in beam profiles for nominal energies of 4 and 10 MV flattened beams. Bending magnet current values were changed by −10% and +10% at the depth of maximum dose $\left(d_{\text{max}}\right)$ and at a depth of 10 cm $\left(d_{10}\right)$ in water. Note that the off‐axis distance of the minimum and maximum change with energy and depth.

For FF beams, we obtained the Flat (Eq. (1)) from the measured profiles for the nominal energy and for energy changes of ±5% and ±10%. Flat was measured with a 3DS in water at $d_{\text{max}}$ and at 10 cm depth and with an ICA at 0.9 cm and 9.9 cm water‐equivalent depths. We calculated changes in flatness from the nominal energy as a function of BMI in water and with the ICA (Table 2). The Flat increased with energy near $d_{\text{max}}$ but decreased with energy near 10 cm depth (Fig. 2(b), Table 3). This reversal in trend is explained by the fact that flat is not defined at a fixed off‐axis distance, and the position of the min and max change with energy and depth (Fig. 3).

We also determined OAR from beam profiles taken along the orthogonal axes for ±5%,±10% BMI changes and at nominal beam energies, both in water and with an ICA. We calculated changes in OAR from the nominal energy as a function of BMI (Table 4). OAR was found to be almost inversely linearly proportional to the changes in BMI (Fig. 2(c)). We compared the results of OAR measured in water near $d_{\text{max}}$ and at depth 10 cm with the corresponding results from using the ICA at similar depths, and we found that the changes in OAR measured in water agreed with those measured with the ICA.

We further found that the changes in OAR were larger than the changes in Flat and showed the same trend with beam energy changes at both depths. The changes in PDD were the smallest compared with OAR and Flat with changes in beam energy. We also evaluated the changes in $F_{\text{DN}}$ from the data measured by using an ICA, because the profiles on the diagonal axes were obtained simultaneously with those on the orthogonal axes. We found that the $F_{\text{DN}}$ defined on the diagonals showed equivalent sensitivity compared with OAR measured on the orthogonal axes.

We calculated the standard deviations of flat and OAR (including $F_{\text{DN}}$ from the ICA data) based on five independent measurements of profile at nominal beam energies for different setup conditions (Table 5). We found that the measurement uncertainty in water was slightly greater than that with the ICA. We also found that, in general, the measurement uncertainty in flat and OARs were equivalent at 0.06%±0.02%(1σ). These uncertainties include setup variations, accelerator performance for a given beam tune, and random errors.

**Table 3:** Changes in flatness, (Flat, %) as a function of bending magnet current (BMI) for 4, 6, 8, and 10 MV flattened beams for a $30\times 30\;{\text{cm}}^{2}$ field

*Measured in water at* $d_{max}$ and $d_{10}=10\;cm$ *depth (averaged in‐plane and cross‐plane)*								
*Change in BMI*	*4 MV*		*6 MV*		*8 MV*		*10 MV*	
	$d_{max}$	$d_{10}$	$d_{\text{max}}$	$d_{10}$	$d_{max}$	$d_{10}$	$d_{max}$	$d_{10}$
10%	−1.2	1.3	−0.5	1.7	−0.2	2.6	−0.6	2.6
5%	−0.6	0.6	−0.9	0.7	−1.0	1.1	−0.9	1.1
0%	0.0	0.0	0.0	0.0	0.0	0.0	0.0	0.0
−5%	1.0	−0.7	1.1	−0.5	1.5	−0.3	1.5	−0.3
−10%	1.9	−0.5	2.4	0.1	3.2	0.8	3.1	0.8
*Measured with an ionization chamber array with no additional buildup (B_0_) or with 9 cm solid water buildup (B_9_)*								
*Change in BMI*	*4 MV*		*6 MV*		*8 MV*		*10 MV*	
	*B*_*0*_	*B*_*9*_	*B*_*0*_	*B*_*9*_	*B*_*0*_	*B*_*9*_	*B*_*0*_	*B*_*9*_
10%	−1.8	1.3	−1.7	1.7	−1.7	2.6	−2.0	1.9
5%	−1.0	0.6	−1.2	0.8	−1.2	1.2	−1.2	0.7
0%	0.0	0.0	0.0	0.0	0.0	0.0	0.0	0.0
−5%	0.8	−0.5	1.1	−0.5	1.5	−0.6	1.3	−0.1
−10%	2.0	−0.8	2.4	−0.3	3.1	0.5	3.0	1.2

**Table 4:** Changes in off‐axis ratio, %, as a function of bending magnet current (BMI) for 4, 6, 8, and 10 MV flattened beams for a $30\times 30\;{\text{cm}}^{2}$ field

*Measured in water at depths of d*_*max*_ *and 10 cm with a fixed off‐axis distance of 80% of field size*								
*Change in BMI*	*4 MV*		*6 MV*		*8 MV*		*10 MV*	
	$d_{max}$	$d_{10}$	$d_{max}$	$d_{10}$	$d_{max}$	$d_{10}$	$d_{max}$	$d_{10}$
10%	−3.9	−2.9	−4.7	−3.8	−5.8	−5.0	−5.6	−5.0
5%	−2.0	−1.5	−2.3	−1.9	−2.9	−2.4	−2.5	−2.2
0%	0.0	0.0	0.0	0.0	0.0	0.0	0.0	0.0
−5%	2.2	1.7	2.7	2.4	3.1	2.6	3.2	3.1
−10%	4.2	3.4	5.3	4.2	6.3	5.4	6.2	6.0
*Measured with an ionization chamber array with no additional buildup (B*_*0*_ *) or with 9 cm solid water buildup (B*_*9*_ *)*								
*Change in BMI*	*4 MV*		*6 MV*		*8 MV*		*10 MV*	
	*B*_*0*_	*B*_*9*_	*B*_*0*_	*B*_*9*_	*B*_*0*_	*B*_*9*_	*B*_*0*_	*B*_*9*_
10%	−4.3	−3.0	−5.1	−4.0	−5.7	−5.0	−5.3	−5.0
5%	−2.1	−1.5	−2.7	−2.0	−2.9	−2.6	−2.5	−2.5
0%	0.0	0.0	0.0	0.0	0.0	0.0	0.0	0.0
−5%	2.4	1.5	2.8	1.8	3.1	2.4	3.2	2.8
−10%	4.6	3.1	5.3	4.0	6.3	5.3	6.3	5.8

**Table 5:** Standard deviation of profile‐based metrics

	*Metric*	*4 MV*	*6 MV*	*8 MV*	*10 MV*
	Water $\left(d_{\text{max}}\right)$	0.07%	0.09%	0.07%	0.05%
Flat	ICA $\left(B_{0}\right)$	0.04%	0.04%	0.04%	0.04%
	Water $\left(d_{10}\right)$	0.07%	0.07%	0.07%	0.06%
	ICA $\left(B_{9}\right)$	0.03%	0.04%	0.03%	0.03%
	Water $\left(d_{\text{max}}\right)$	0.09%	0.12%	0.09%	0.05%
	ICA $\left(B_{0}\right)$	0.07%	0.09%	0.07%	0.05%
Off‐axis ratio	ICA $\left(F_{\text{DN}},B_{0}\right)$	0.06%	0.08%	0.06%	0.05%
	Water $\left(d_{10}\right)$	0.09%	0.11%	0.09%	0.06%
	ICA $\left(B_{9}\right)$	0.05%	0.06%	0.05%	0.04%
	ICA $\left(F_{\text{DN}},B_{9}\right)$	0.07%	0.07%	0.07%	0.07%

Flat, off‐axis ratio, and diagonal normalized flatness $\left(F_{\text{DN}}\right)$ were measured using an ionization chamber array (ICA) without buildup $\left(B_{0}\right)$ and with 9 cm buildup $\left(B_{9}\right)$; flatness and off‐axis ratio were also measured in water at $d_{\text{max}}$ and depth 10 cm $\left(d_{10}\right)$.

#### B.2 Flattening filter‐free beams

**Figure 4:**
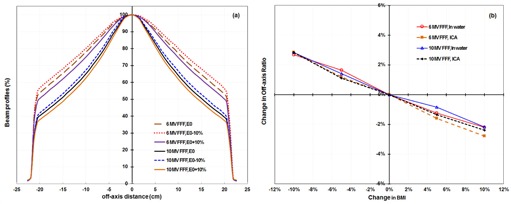
Changes in a diagonal 6 MV and 10 MV flattening filter‐free (FFF) beam profile (a) as a function of energy, measured with an ionization chamber array for a $30\times 30\;{\text{cm}}^{2}$ field. (b) Changes in off‐axis ratio as a function of bending magnet current (BMI) for 6 MV or 10 MV FFF beams for a $30\times 30\;{\text{cm}}^{2}$ field.

For FFF beams, the measured profiles showed that when the beam energy was increased, the profile became more forward‐peaked (Fig. 4(a)). We determined the OAR from the beam profiles for ±5% and ±10% BMI changes and at nominal beam energies, both in water at 10 cm depth and with an ICA at 0.9 cm equivalent water depth. We calculated the changes in OAR from the nominal energy vs. BMI (Table 6). Unfortunately, we did not obtain data at equivalent depths with the ICA and water during this part of this study, making direct comparisons difficult. The changes in OAR were nearly linear, with energy variation over the range of BMI values from −10% to +10% (Fig. 4(b)).

We also estimated the measurement uncertainty in OAR and $F_{\text{DN}}$ (ICA) data based on five independent measurements of profile at nominal beam energies. As was true for the FF beams, these uncertainties included setup variations, accelerator performance for a given beam tune, and random errors and are listed in Table 7.

**Table 6:** Changes in off‐axis ratio, %, as a function of bending magnet current (BMI) for 6 and 10 MV flattening filter‐free beams for a $30\times 30\;{\text{cm}}^{2}$ field

*Change in BMI*	*6 MV FFF*		*10 MV FFF*	
	*ICA* $\left(B_{0}\right)$	*Water* $\left(d_{10}\right)$	*ICA* $\left(B_{0}\right)$	*Water* $\left(d_{10}\right)$
10%	−3.1	−2.2	−2.2	−2.2
5%	−2.0	−1.2	−1.4	−0.8
0%	0.0	0.0	0.0	0.0
−5%	0.9	1.7	0.7	1.4
−10%	2.5	2.7	2.1	2.8

$d_{10}=$ dose at depth of 10 cm, measured in water; $B_{0}=$ dose measured with an ionization chamber array (ICA) with no additional buildup.

**Table 7:** Standard deviations of off‐axis ratio and $F_{\text{DN}}$ for flattening filter‐free (FFF) beams

*Off‐axis Ratio*	*6 MV FFF*	*10 MV FFF*
Water $\left(d_{10}\right)$	0.08%	0.08%
ICA $\left(B_{0}\right)$	0.05%	0.04%
ICA $\left(F_{\text{DN}},B_{0}\right)$	0.05%	0.03%

$d_{10}=$ dose at depth of 10 cm, measured in water; $B_{0}=$ dose measured with an ionization chamber array (ICA) with no additional buildup; $F_{\text{DN}}=$ diagonal normalized flatness.

### C. Minimum detectable changes in energy

#### C.1 Flatting filter beams

We estimated the minimum detectable energy change for PDD(10), Flat, OAR, and $F_{\text{DN}}$ metrics by using a 95% confidence level (2σ). First we fit a linear equation to the change in each metric to the change in energy; we included only data for energy changes up to ±5% from the nominal value because this range covers the minimum detectable change. Then we used the linear equation to determine what energy change would be needed to produce a 2σ change in each metric for all FF beams (Table 8).

**Table 8:** Minimum detectable change in energy, %, of various metrics for flattening filter beams

	*Metric*	*4 MV*	*6 MV*	*8 MV*	*10 MV*
	PDD(10)	2.4%	2.3%	2.7%	2.5%
		(1.9%)^a^	(1.8%)^a^	(2.2%)^a^	(2.1%)^a^
	water $\left(d_{\text{max}}\right)$	0.9%	0.9%	0.6%	0.6%
	ICA $\left(B_{0}\right)$	0.5%	0.4%	0.4%	0.4%
Flatness	water $\left(d_{10}\right)$	1.4%	1.5%	1.7%	1.7%
	ICA $\left(B_{9}\right)$	0.7%	0.7%	0.6%	0.6%
	Water $\left(d_{\text{max}}\right)$	0.4%	0.5%	0.3%	0.3%
	ICA $\left(B_{0}\right)$	0.3%	0.3%	0.2%	0.2%
	ICA $\left(F_{\text{DN}},B_{0}\right)$	0.2%	0.2%	0.2%	0.2%
Off‐Axis Ratio	Water $\left(d_{10}\right)$	0.5%	0.5%	0.4%	0.3%
	ICA $\left(B_{9}\right)$	0.3%	0.3%	0.2%	0.2%
	ICA $\left(F_{\text{DN}},B_{9}\right)$	0.3%	0.2%	0.3%	0.3%

a Numbers in parentheses are PDD(10) of an $5\times 5\;{\text{cm}}^{2}$ field.

$d_{10}=$ dose at depth of 10 cm, measured in water; $B_{0}=$ dose measured with an ionization chamber array (ICA) with no additional buildup; $F_{\text{DN}}=$ diagonal normalized flatness.

#### C.2 Flattening filter‐free beams

For FFF beams, we followed the same procedure as for the FF beams to determine the minimum detectable energy change at a 95% confidence level (Table 9).

**Table 9:** Minimum detectable change in energy, %, for flattening filter‐free (FFF) beams

	**Metric**	*6 MV FFF*	*10 MV FFF*
	PDD(10)	2.4% (1.9%)^a^	2.30% (1.8%)^a^
	water $\left(d_{10}\right)$	0.5%	0.6%
Off‐Axis Ratio	ICA $\left(B_{0}\right)$	0.3%	0.3%
	ICA $\left(F_{\text{DN}},B_{0}\right)$	0.3%	0.3%

a Numbers in parentheses are PDD(10) of an $5\times 5\;{\text{cm}}^{2}$ field.

## IV. DISCUSSION

Two factors affect the detection of the minimal change in energy: 1) the sensitivity of the metric to change in energy, and 2) the uncertainty in the measurements. Percent depth dose at 10 cm depth, PDD(10), was found to be monotonic with energy change, with the greatest changes occurring for the smallest field sizes (Fig. 2(a)). Changes in PDD(10) between the smallest field size studied $\left(5\times 5\;{\text{cm}}^{2}\right)$ and the $10\times 10\;{\text{cm}}^{2}$ field tabulated in this work (Table 1) were at most 0.5%. The $10\times 10\;{\text{cm}}^{2}$ field is the most often used in clinical settings. The minimum detectable change when using PDD(10) was approximately 2.5% (Table 7) with a $10\times 10\;{\text{cm}}^{2}$ field, which is used routinely for monthly QA. This 2.5% variation in energy could lead to changes in OAR that exceed the 1% tolerance specified in the American Association of Physicists in Medicine recommendations for linac QA.^(1)^ The minimum detectable change in energy could be reduced to 1.8%–2.1% by using a $5\times 5\;{\text{cm}}^{2}$ field (Tables 8 and 9), but this is still much larger than the minimum detectable energy change using any of the other profile‐based metrics studied.

Flat was not the most sensitive of the metrics studied and was the only metric that showed a nonmonotonic behavior with energy changes. Flat was also unique in showing a near inversion in its relationship with energy change when measured near $d_{\text{max}}$ versus measured at 10 cm depth (Fig. 2(b)). The Flat metric was able to detect changes in energy of <1% if measured at a depth near $d_{\text{max}}$ but would be approximately 1.5% if measured at a depth near 10 cm. These observations lead us to believe that Flat is not the best metric for determining changes in energy.

Off‐axis, ratio‐based metrics showed monotonic behavior regardless of depth. They showed good sensitivity for all beams and had low measurement uncertainty whether measured in water or with an ICA. Changes in $\text{OAR}/F_{\text{DN}}$‐based metrics were not always significantly larger than changes in PDD(10), but the uncertainty in the measurement of the $\text{OAR}/F_{\text{DN}}$ was always lower than that in the PDD. $\text{OAR}/F_{\text{DN}}$ metrics were able to detect very small changes in energy (i.e., only ±5%). We found equivalent sensitivity regardless of whether the measurements were made on the orthogonal axes (OAR) or on the diagonals $\left(F_{\text{DN}}\right)$.

All profile‐based energy metrics in this study were examined by using both a 3D water scanner and a 2D planner array. The derived metrics from these devices were very similar, and both were highly predictive of energy changes for both FF and FFF beams. For routine QA, a planner array is more convenient than a water tank, and the measurement reproducibility of ICA is slightly better than that of the water tank scanning system (Table 5). Our findings suggest that the data derived from an ICA are equally sensitive for detecting energy changes as data derived from a water scanning system.

## V. CONCLUSIONS

Of the metrics studied, the off‐axis ratio‐based metrics (OAR or $F_{\text{DN}}$) were best able to detect an energy change for both FF and FFF beams. This energy change could be measured by using either a water scanner or a 2D planner array. The 2D array has an added advantage, as it is generally used for other monthly performance tests of the linac such as beam symmetry verification, and because the setup is much easier than that for the water scanning system. We recommend the measurement of off‐axis ratio either on the orthogonal axes or the diagonals, using an ICA near the depth of the maximum dose, as a sensitive and efficient method to confirm the stability of photon beam energy.

## ACKNOWLEDGMENTS

The authors thank Mike Tham, Daniel Dingbaum, Rogerio Silveira (Varian), and Johnnie Nowells (Sun Nuclear) for engineering support during the measurements. We thank Michael Gillin and Ramaswamy Sadagopan for help in reviewing this manuscript. We also thank Christine Wogan for scientific editing of this manuscript.

## COPYRIGHT

This work is licensed under a Creative Commons Attribution 3.0 Unported License.
